# Effect of perioperative autonomic nervous system imbalance on surgical outcomes: a systematic review

**DOI:** 10.1016/j.bja.2025.06.004

**Published:** 2025-07-04

**Authors:** Wei-Tong Pan, Mu-huo Ji, Daqing Ma, Jian-Jun Yang

**Affiliations:** 1 Department of Anesthesiology, Pain and Perioperative Medicine, The First Affiliated Hospital of Zhengzhou University, Zhengzhou, Henan, China; 2 Neuroscience Research Institute, Zhengzhou University Academy of Medical Sciences, Zhengzhou, Henan, China; 3 Department of Anesthesiology, The Second Affiliated Hospital of Nanjing Medical University, Nanjing, Jiangsu, China; 4 Perioperative and Systems Medicine Laboratory and Department of Anesthesiology, Children’s Hospital, Zhejiang University School of Medicine, National Clinical Research Center for Child Health, Hangzhou, Zhejiang, China; 5 Department of Anesthesiology, The First Affiliated Hospital, Ningbo University, Ningbo, Zhejiang, China; 6 Division of Anaesthetics, Pain Medicine & Intensive Care, Department of Surgery & Cancer, Faculty of Medicine, Imperial College London, Chelsea & Westminster Hospital, London, UK

**Keywords:** autonomic nervous system, perioperative autonomic imbalance, parasympathetic tone, perioperative outcomes, vagus nerve stimulation

## Abstract

The autonomic nervous system (ANS) is essential for maintaining physiological homeostasis. Autonomic nervous system imbalance, characterised by sympathetic hyperactivation and low parasympathetic tone, can occur during the perioperative period. These changes drive systemic stress responses, cardiovascular instability, impaired tissue repair, and immunosuppression, which in turn increase infection risk, neurocognitive decline, and multiorgan dysfunction. Surgical trauma, anaesthesia, pain, hypothermia, and psychological stressors all contribute to this dysregulation, and consequently low parasympathetic tone results in the cholinergic anti-inflammatory pathway being less effective. High sympathetic nervous system activity promotes catecholamine surges and pro-inflammatory cytokine release. Pharmacological interventions, including dexmedetomidine and β-blockers, together with nonpharmacological strategies, such as electroacupuncture and temperature management, are measures that have potential to restore ANS balance. This systematic review covers ANS-mediated organ regulation, pathophysiological consequences of perioperative dysautonomia, and evidence-based therapeutic strategies. By integrating findings from multiple basic and clinical studies, the pivotal roles of ANS modulation in mitigating postoperative complications, including neurocognitive disorders, immunosuppression, and cancer recurrence, are discussed. Maintaining balance of the sympathetic and parasympathetic nervous systems is an important prospect in perioperative medicine that could benefit surgical patients’ short- or long-term recovery.

**Editor’s key points**

• Perioperative autonomic nervous system (ANS) imbalance occurs frequently, but its consequences remain elusive.
• This systematic review discusses the possible contributions of perioperative ANS imbalance to perioperative complications, and potential pharmacological and nonpharmacological interventions to treat this imbalance.
• Restoring ANS balance offers a promising strategy to mitigate surgical complications and improve recovery. Optimising personalised interventions to treat ANS imbalance could enhance surgical outcomes and warrants further study.

The autonomic nervous system (ANS), with the sympathetic (SNS) and parasympathetic (PSNS) nervous systems, orchestrates cardiorespiratory stability, immune response, and metabolic processes via complex neuroanatomical and neurochemical interactions.^1^ Surgical stress, low blood volume, pain, hypothermia, and psychological stress enhance sympathetic activity, leading to increased catecholamine release, tachycardia, and pro-inflammatory cytokines.^2^, ^3^, ^4^, ^5^, ^6^, ^7^ Simultaneously, reduced parasympathetic activity compromises cholinergic anti-inflammatory mechanisms, further disrupting immune and metabolic homeostasis. These perturbations are not simply transient, but can even lead to postoperative complications and poor long-term outcomes, especially in populations with pre-existing diseases.^8^

A characteristic feature of perioperative ANS dysfunction is a significant reduction of parasympathetic tone, which disturbs homeostasis and triggers a cascade of adverse outcomes. This imbalance exacerbates systemic stress responses and impairs tissue repair, thereby increasing susceptibility to postoperative neurocognitive disorders (PND), infection, cancer reoccurrence, or even multiorgan dysfunction.^9^, ^10^, ^11^ Contemporary perioperative strategies for ANS modulation, such as dexmedetomidine and β-blockers, electroacupuncture, and temperature and exercise management, show promise in mitigating adverse effects after surgery and enhancing patient recovery.

We conducted a literature search strategy in accordance with PRISMA guidelines (Supplementary material and Supplementary Fig. 1) to build this systematic review. The aims of this review are to integrate the critical function of the ANS in perioperative physiology, elucidate the pathophysiological implications of its dysregulation, and assess evidence-based strategies for restoring ANS balance. By synthesising insights from the fields of anaesthesia and neuroscience, we seek to underscore the potential of ANS-targeted interventions in enhancing patient care and recovery after surgery.

## Autonomic nervous system and homeostasis

**Fig 1:**
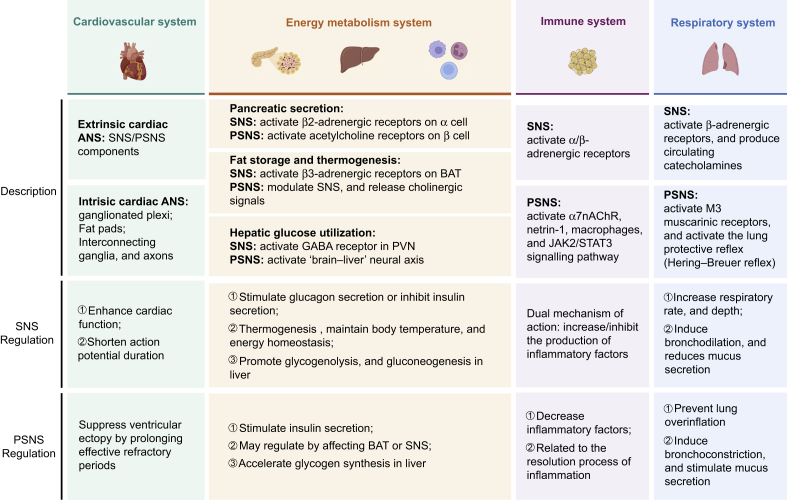
Autonomic nervous system (ANS) regulates specific molecules, pathways, and effects produced in various organs. BAT, brown adipose tissue; GABA, γ-aminobutyric acid; JAK, Janus kinase; PSNS, parasympathetic nervous system; PVN, paraventricular nucleus; SNS, sympathetic nervous system; STAT, signal transducer and activator of transcription. Created with elements from https://BioRender.com.

The ANS is the primary neural basis for maintaining the physiological balance of organ systems, dynamically balancing SNS and PSNS output to adapt to internal and external stressors. This dual innervation is achieved through precise neuroanatomical circuits, neurotransmitters, cellular signals, and receptors, ensuring the coordination and stability of cardiovascular, metabolic, immune, and respiratory functions (Fig. 1).

### Cardiovascular system

The ANS achieves precise cardiovascular regulation through antagonism between the SNS and the PSNS. Functionally, SNS activation enhances cardiac function through β1-mediated increases of heart rate (chronotropy), contractility (inotropy), and atrioventricular conduction velocity (dromotropy) while shortening ventricular action potential duration.^12^ Sympathetic hyperactivation shortens the ventricular action potential duration but increases transmural dispersion of repolarisation, predisposing to arrhythmias in conditions such as heart failure. In contrast, parasympathetic hyperactivation suppresses ventricular ectopy by prolonging effective refractory periods, but it may enhance atrial arrhythmia through heterogeneous repolarisation.^13^ In conclusion, the ANS does not maintain cardiovascular homeostasis through static control but rather regulates the continuous rebalancing of SNS and PSNS at the molecular, cellular, and systemic levels.

### Energy metabolism system

Energy balance, or energy homeostasis, involves the CNS receiving and integrating peripheral energy signals. The ANS governs energy homeostasis through bidirectional communication between hypothalamic nuclei and peripheral metabolic organs, including the pancreas, adipose tissue, and liver.

#### Pancreatic secretion

The ANS regulates islet cells through neurotransmitters and hormones, which respond to changes in blood glucose to maintain its concentration within the normal range.^14^ The SNS plays a significant role in stimulating glucagon secretion or inhibiting insulin secretion by activating β2-adrenergic receptors on α cells, whereas PSNS stimulation promotes insulin secretion by activating acetylcholine (ACh) receptors in β cells.^15^

#### Fat storage and thermogenesis

The ANS can affect energy balance and thermogenesis by regulating adipose tissue metabolism. Postganglionic sympathetic fibres release norepinephrine to bind β3-adrenergic receptors on brown adipose tissue adipocytes, initiating a cAMP-PKA signalling cascade that activates uncoupling protein 1 (UCP1) to generate heat.^16^ Concurrently, SNS activation promotes lipolysis, ensuring substrate availability for energy expenditure. In contrast, PSNS contributions to adipose metabolism remain less defined but it may be involved in dual mechanisms: indirect modulation of SNS activity through central circuits or direct cholinergic signalling to adipocytes.^17^

#### Hepatic glucose utilisation and glycogen synthesis

The ANS can control liver metabolism through neural pathways and promote overall energy balance. A previous study reported that the glucose-lowering effects induced by insulin administration to the brain via the carotid artery persisted even after removal of the pancreas and gastrointestinal tract, suggesting the existence of a separate ‘brain–liver’ neural axis.^18^ The ANS can finely regulate the metabolic activity of the liver, promoting glycogenolysis and gluconeogenesis to provide energy by releasing catecholamines such as epinephrine and norepinephrine.^19^

### Immune system

The SNS modulates immune responses via norepinephrine release, which binds β2-adrenergic receptors on macrophages and glial cells to suppress pro-inflammatory cytokines (e.g. tumour necrosis factor-alpha [TNF-α], interleukin-6 [IL-6]) by inhibiting nuclear factor kappa B (NF-κB) signaling^20^ while simultaneously inducing anti-inflammatory mediators such as IL-10.^21^ Notably, β2-receptor stimulation can also prevent synaptic dysfunction and neurotoxicity by increasing brain-derived neurotrophic factor (BDNF) and alleviating the inflammatory effect, highlighting SNS’s dual immunomodulatory and neural repair functions.^22^^,^^23^ In addition, the SNS also acts on monocytes and macrophages via α-adrenergic receptors, possibly leading to an increase of pro-inflammatory cytokines.^24^ This enables dynamic immune adaptation to physiological or pathological demands. Conversely, the PSNS counterbalances SNS activity through the cholinergic anti-inflammatory pathway by vagal efferent release of ACh to activate α7 nicotinic receptors (α7nAChR) on immune cells. This subsequently suppresses NF-κB-driven inflammation while activating JAK2/STAT3 signalling to resolve inflammation.^25^ This pathway enhances macrophage phagocytosis and upregulates netrin-1, facilitating the transition from acute inflammation to tissue repair.^26^^,^^27^

### Respiratory system

The regulation of the respiratory system by the ANS is achieved through the integration of sensory input and motor output within the brainstem. Hypoxia and hypercapnia trigger SNS responses that increase respiratory rate and depth, optimising gas exchange.^28^ Sensory afferents, primarily carried by the PSNS, detect mechanical and chemical stimuli in the airways, activating reflexes such as the Hering–Breuer reflex to prevent lung overinflation or initiating protective responses such as coughing and bronchoconstriction via pulmonary stretch receptors, rapidly adapting receptors, and bronchial and pulmonary C-fibre terminals.^29^ In addition, the ANS also can directly regulate movement and mucus secretion of bronchi to affect respiration. The PSNS dominates baseline airway tone through vagal efferent release of ACh, acting on M3 muscarinic receptors to induce bronchoconstriction and stimulate mucus secretion.^30^ In contrast, the SNS induces bronchodilation and reduces mucus secretion primarily via circulating catecholamines.^31^ This dynamic balance between the PSNS and the SNS ensures efficient ventilation–perfusion matching and airway protection under normal conditions, and this balance can be profoundly affected during the perioperative period.

## Perioperative pathophysiology and risk factors for autonomic nervous system imbalance

The ANS balance can be disrupted through multifactorial mechanisms including surgical trauma, haemodynamic instability, nociceptive signalling, thermoregulatory challenges, and psychological stress acting as the key drivers of sympathetic hyperactivation and parasympathetic suppression during the perioperative period. These perturbations create a maladaptive neuroendocrine milieu that exacerbates systemic inflammation, compromises organ perfusion, and increases susceptibility to postoperative complication occurrence.

### Surgery, surgical trauma, and stress

Surgical trauma initiates a dual-axis neuroendocrine stress response through activation of the hypothalamic–pituitary–adrenal and sympathetic–adrenal–medullary axes.^2^ Clinically, chronic sympathetic overdrive promotes endothelial dysfunction via reduced nitric oxide bioavailability, enhanced vascular permeability and prothrombotic states, and increases myocardial ischaemia, arrhythmia, and heart failure risks.^32^ Correspondingly, parasympathetic suppression may impair inflammation resolution and tissue repair mechanisms, predisposing to chronic pain and impaired wound healing.^33^ Prolonged and major surgery can further aggravate systemic inflammation and ANS imbalance.^34^ Open procedures induce prolonged inflammatory states and remarkable ANS imbalance as a result of extensive tissue trauma, whereas minimally invasive surgery (e.g. laparoscopy) preserves heart rate variability (HRV) with less sympathetic hyperactivity and stress responses.^3^^,^^35^ Thus, unnecessary major surgery should be always avoided in clinical practice.

### Low blood volume

Low blood volume owing to haemorrhage can trigger a significant sympathetic response to maintain perfusion pressure, leading to tachycardia and vasoconstriction. Excessive fluid administration can lead to parasympathetic activation to counteract volume overload, causing bradycardia and hypotension.^36^ Precise intraoperative management of blood volume is crucial in maintaining ANS stability and preventing complications. Monitoring blood volume and ensuring adequate fluid load can prevent issues such as hypovolaemia or fluid overload, both of which can adversely impact patient outcomes.^6^

### Pain

Pain leads to SNS overactivation while suppressing PSNS activity, causing dysregulation of the ANS. The ongoing dysfunction of the ANS results in chronic pain syndromes.^37^ Persistent pain can further exacerbate ANS dysfunction, forming a vicious cycle. By regulating the activity of the PSNS, the autonomic function of patients can be improved, which may reduce their pain and enhance postoperative recovery.^8^ However, this area of research is limited.

### Intraoperative hypothermia

Intraoperative hypothermia, defined as a core temperature below 36°C, is common and can impact the ANS, leading to various physiological disturbances.^5^ Firstly, hypothermia can affect the balance of the SNS and PSNS, causing haemodynamic instability and potassium ion metabolism disorder, and increasing cardiovascular complication risk.^38^ Secondly, hypothermia may affect immune function, increase the risk of postoperative infection risk, and lead to delayed postoperative recovery.^39^ Therefore, it can be inferred that hypothermia may affect prognosis by affecting ANS function.

### Fear, depression, and anxiety

Preoperative fear, depression, and anxiety are common psychological states in the perioperative period, and these emotional factors may lead to ANS dysfunction.^40^ Clinical studies showed that patients with preoperative depression and anxiety had lower HRV, indicating enhanced SNS activity and reduced PSNS activity.^7^ This ANS dysregulation led to poor postoperative recovery and increased the risk of postoperative complications. Mechanistically, SNS overactivity exacerbated preoperative depression and anxiety through activating astrocytes in the anterior cingulate cortex,^41^ although other mechanisms may be also involved.

## Perioperative autonomic nervous system function assessment

**Fig 2:**
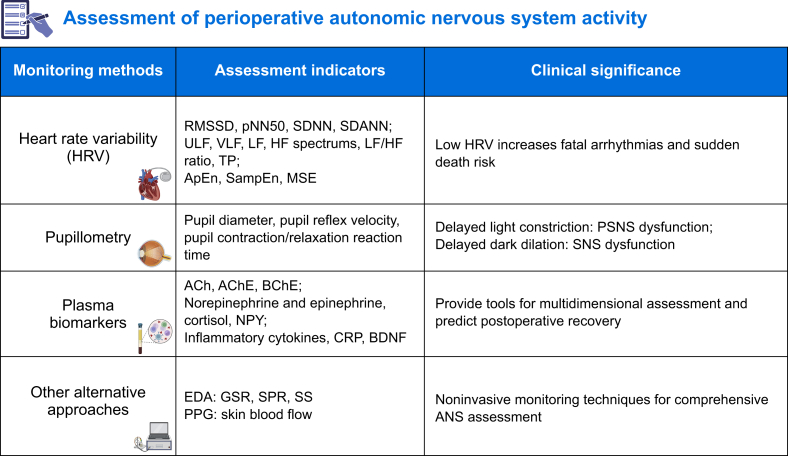
Perioperative assessment methods, indicators, and clinical significance of autonomic nerves system (ANS) activity. These indicators respond and ANS function to varying degrees. ACh, acetylcholine; AChE, acetylcholinesterase; BChE, butyrylcholinesterase; BDNF, brain-derived neurotrophic factor; CRP, C-reactive protein; EDA, electrodermal activity; GSR, galvanic skin response; HF, high-frequency spectrums; LF, low frequency; NPY, neuropeptide Y; pNN50, the percentage of successive normal sinus RR intervals >50 ms; PPG, photoplethysmography; PSNS, parasympathetic nervous system; RMSSD, the root mean square of the successive normal sinus RR interval difference; SDANN, the standard deviation of the averaged normal sinus RR intervals for all 5-min segments; SDNN, the standard deviation of all normal sinus RR intervals during 24 h; SNS, sympathetic nervous system; SP, skin potential; SS, skin susceptance; TP, total power; ULF, ultra-low frequency; VLF, very low frequency. Created with elements from https://BioRender.com.

There is no ‘gold standard’ to monitor ANS function, but several complementary indictors can be detected simultaneously in clinical settings. These include HRV, pupillometry, blood biomarkers, skin conductance level, and other indicators (Fig. 2).

### Heart rate variability

Real-time fluctuations in heartbeat intervals are reflected by HRV and are regulated by the ANS through its influence on the sinoatrial node.^42^ Healthy hearts generally have high HRV, whereas low HRV increases the likelihood of fatal arrhythmias and sudden death.^43^ Transient or short-term changes in the cardiac cycle are associated with respiratory movements and can reflect parasympathetic activity, whereas long-term changes are more indicative of sympathetic function.^44^ Noninvasive HRV analysis evaluates ANS balance by assessing heartbeat interval (RR interval) dynamics through time-domain, frequency-domain, and nonlinear measurement (Fig. 2).^45^ Time-domain indicators such as the standard deviation of RR intervals (SDNN) and root mean square of successive differences (RMSSD) quantify overall variability and parasympathetic modulation, with SDNN <50 ms indicating potential ANS dysfunction. Frequency-domain analysis decomposes HRV into spectral bands: the low-frequency (LF) spectrum (0.04–0.15 Hz) reflects combined SNS and PSNS activity, whereas the high-frequency (HF) spectrum (0.16–0.40 Hz) predominantly measures PSNS activity. The LF/HF ratio estimates SNS and PSNS balance but requires cautious interpretation owing to other influencing factors such as age and circadian rhythms. Total spectral power represents overall ANS modulation, with declines suggesting attenuation of ANS activity.^46^ Nonlinear methods, such as entropy measures (ApEn, SampEn) and multiscale entropy (MSE), capture the complexity and unpredictability of HRV, offering different ways to monitor the dynamic changes of ANS activity in surgical patients during stress.^47^ Together, these methods for assessing ANS function are helpful for perioperative risk stratification and monitoring physiological responses.

### Pupillometry

Pupillary response can reflect the neurophysiological state of the patient and is a vital tool for assessing the balance of the ANS activity. Based on the pupil light response, delayed pupil constriction in light suggests PSNS dysfunction, whereas delayed pupil dilation in dark indicates SNS dysfunction.^48^ Perioperatively, pupillometry aids in monitoring ANS function, assessing the depth of anaesthesia, and guiding drug dosing adjustments, particularly in ophthalmic procedures.^49^ However, factors such as ambient light, individual differences, and external interference limit its reliability as an independent assessment tool for ANS activity.

### Biomarkers of parasympathetic function

#### Acetylcholine

As a primary neurotransmitter of the PSNS, ACh modulates cardiovascular, respiratory, and digestive functions.^50^ Monitoring of plasma ACh concentrations helps to monitor parasympathetic tone,^51^ and small changes can be reflected in HRV values. Additionally, emerging techniques, including real-time electrochemical biosensors and microdialysis coupled with mass spectrometry, now allow for dynamic perioperative ACh monitoring.^52^

#### Cholinesterase activity

The activity of cholinesterase, including acetylcholinesterase (AChE) and butyrylcholinesterase (BChE), is another critical indicator of vagal function. Perioperative decreases in cholinesterase activity have been linked to prolonged neuromuscular block and increased susceptibility to infections as a result of impaired immune function.^53^ Biosensors to detect cholinesterase activity have been developed,^54^ highlighting their potential as a diagnostic tool for perioperative ANS tone.

### Biomarkers of sympathetic function

#### Norepinephrine and epinephrine

Elevated concentrations of these catecholamines are associated with increased perioperative morbidity, including hypertension, arrhythmias, and myocardial ischaemia.^55^ Recent advances in continuous *in vivo* monitoring, such as enzyme-based electrochemical sensors and minimally invasive microdialysis probes, enable real-time measurement of circulating catecholamines. Additionally, noninvasive approaches such as sweat-based wearable biosensors (e.g. epinephrine/dopamine detecting patches) offer clinically feasible alternatives for dynamic perioperative assessment.^56^

#### Cortisol

Perioperative cortisol concentrations increase as part of the stress response, with elevated concentrations correlating with increased surgical stress and postoperative complications such as impaired wound healing and infections.^57^ Previous studies showed that saliva and sweat monitoring of cortisol concentrations can be used as a noninvasive method to evaluate perioperative ANS tone,^52^^,^^58^ but further research is needed.

#### Neuropeptide Y

Elevated perioperative concentrations of neuropeptide Y (NPY), a co-transmitter released with norepinephrine from sympathetic neurones that modulates vasoconstriction and stress responses, reflect heightened sympathetic activity and are associated with adverse cardiovascular events.^59^ NPY concentrations can be monitored by plasma and sweat, combined with the dynamic changes of HRV, which can improve the accuracy of perioperative assessment of ANS function.^52^

### Other biomarkers

#### Inflammatory cytokines

Pro-inflammatory cytokines such as IL-6 and TNF-α are critical mediators of the inflammatory response. Elevated concentrations of these cytokines perioperatively indicate a heightened sympathetic response and are predictive of complications such as sepsis and prolonged hospital stays.^60^ Some wearable impedance sensors can detect various inflammatory markers in sweat, which is helpful for monitoring of autonomic tone.^52^

#### C-reactive protein

As a well-established biomarker of systemic inflammation and autonomic dysregulation, C-reactive protein (CRP) exhibits perioperative clinical significance through its association with surgical stress and postoperative complications such as infections and cardiovascular events.^61^ Studies have shown that the microfluidic chip combined with sweat sensor can realise real-time monitoring of CRP, which is helpful for the assessment of perioperative ANS tone.^52^

#### Brain-derived neurotrophic factor

As a neurotrophic factor that is critical for neuronal growth and plasticity, BDNF has its secretion controlled by the ANS.^62^ Perioperative BDNF concentrations can reflect the extent of neural stress and injury, with lower concentrations being associated with poor cognitive outcomes after surgery.^63^ Emerging techniques including ultrasensitive single molecule assays and real-time microdialysis enable precise BDNF measurement.

### Other alternative measurement

#### Electrodermal activity

The main components of electrodermal activity (EDA) include galvanic skin response, skin potential, and skin susceptance, and their values are primarily determined by the activity of eccrine sweat glands, which are regulated by the SNS.^64^ Studies showed that patients with high preoperative EDA concentrations experienced high intensity pain and prolonged recovery times after surgery.^65^ Modern EDA monitoring technology, such as portable wearable EDA sensors which can achieve real-time and continuous monitoring, has made it more convenient and increased its use.^66^

#### Photoplethysmography

By detecting changes in skin blood flow, photoplethysmography (PPG) serves as a noninvasive technique for assessing cardiovascular activity and ANS function, which enables real-time monitoring of heart rate, oxygen saturation, and pulse wave transit time to assist in anaesthesia dose adjustment and haemodynamic management during surgery.^67^ Combined with electrocardiogram and blood pressure, PPG facilitates perioperative ANS assessment and clinical decision.

## Harmful postoperative autonomic nervous system activity imbalance

**Fig 3:**
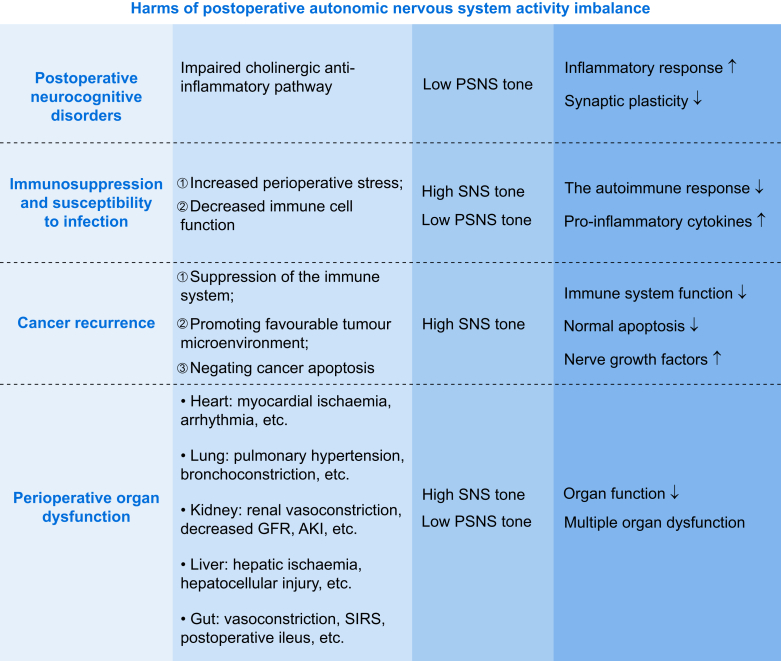
An imbalance in autonomic nervous system (ANS) activity after surgery can hinder recovery and increase the risk of neurocognitive disorders, infections, and other complications. AKI, acute kidney injury; GFR, glomerular filtration rate; PSNS, parasympathetic nervous system; SIRS, systemic inflammatory response syndrome; SNS, sympathetic nervous system. Created with elements from https://BioRender.com.

Perioperative ANS dysfunction can lead to neurocognitive disorders and immunosuppression, increase infection risk, promote cancer recurrence and metastasis, and can even develop into multiple organ dysfunction syndrome (MODS). The mechanism involves uncontrolled inflammation, immunosuppression, tumour microenvironment remodelling, and organ blood flow/metabolic disorders, further highlighting the key effect of perioperative ANS regulation on patient prognosis (Fig. 3).

### Postoperative neurocognitive disorders

Epidemiological studies showed that PND occur in up to 65% of older surgical patients, and the incidence is higher in the older patients after major surgery.^68^ Low parasympathetic tone activity is associated with stress responses that may adversely affect cognitive function. Indeed, lower HRV, a proxy for parasympathetic activity, was associated with the development of PND.^11^ Conversely, strategies aimed at bolstering parasympathetic tone showed potential in mitigating PND risk.^9^

The inflammatory response after surgery is a well-established factor contributing to PND.^69^ The cholinergic anti-inflammatory pathway modulated by the PSNS can attenuate this response. Activation of α7nAChR on macrophages and other immune cells by ACh leads to suppression of pro-inflammatory cytokine release.^70^ Hence, low parasympathetic tone may reduce cholinergic signalling, resulting in heightened and prolonged inflammatory responses, thereby increasing the risk of PND. Animal studies also demonstrated the potential therapeutic role of vagus nerve stimulation (VNS) in PND.^71^ In contrast, the parasympathetic neurotransmitter ACh maintains learning and memory function by enhancing the efficiency of synaptic transmission. Surgical stress and associated anaesthetics can disrupt cholinergic transmission, which may impair synaptic plasticity and lead to cognitive deficits.^72^ Furthermore, the cholinergic system regulates cerebral blood flow, and reductions in parasympathetic tone compromise the delivery of oxygen and nutrients to the brain, exacerbating neuronal dysfunction.^73^ This highlights the importance of parasympathetic modulation of neuronal function in the context of PND.

### Immunosuppression and susceptibility to infection

Surgical trauma is a significant stressor that activates a complex immune response. This immune response can lead to perioperative immunosuppression or an increased risk of postoperative infection, affecting up to 30% of older surgical patients.^74^ Perioperative stress may affect ANS function, especially parasympathetic hypotonia, which may lead to the development of immunosuppression and result in infectious complications.

The SNS plays a crucial role in coping with traumatic events such as surgery. The continuous activation of SNS may also weaken the autoimmune response, thereby reducing the body's defence ability against infection. For example, an experimental study demonstrated that the stimulation of SNS in animals can suppress the phagocytic function of immune cells, such as macrophages.^75^ In contrast to the SNS, the PSNS can promote the activity of immune cells and protect the body from systemic inflammation by releasing ACh, termed the ‘cholinergic anti-inflammatory pathway’.^76^ Animal experiments also showed that by increasing parasympathetic tone, the proliferation and activation of immune cells such as lymphocytes can be seen, indicating that the activation of PSNS can enhance the immune response.^77^ However, PSNS activity may be reduced during postoperative recovery, leading to decreased immune cell function. This imbalance may make patients more susceptible to infection, especially during hospitalisation where there is a high risk of infection.

### Cancer recurrence

Surgery is a frontline treatment for a variety of cancers and can lead to an imbalance in the ANS function, increasing the risk of cancer recurrence and metastasis.^10^ The effect of perioperative ANS imbalance on cancer recurrence is mainly caused by but not limited to: (1) suppression of the immune system; (2) promoting favourable tumour microenvironment; and (3) negating cancer cell apoptosis. SNS activation triggers the release of norepinephrine and epinephrine and binds to β-adrenergic receptors on tumour cells and stromal components, enhancing vascular permeability and extracellular matrix degradation to promote metastatic spread.^78^^,^^79^ Clinical studies corroborated these findings, showing that elevated postoperative catecholamine concentrations correlated with increased circulating tumour cells and reduced survival of different cancer types.^79^ Parasympathetic withdrawal impairs the cholinergic anti-inflammatory pathway, a critical regulator of tumour surveillance. Vagal efferent signalling via α7nAChR suppresses pro-inflammatory cytokines (e.g. IL, TNF-α) and may polarise macrophages towards an anti-tumour M1 phenotype.^80^ Perioperative loss of this modulation creates a pro-inflammatory milieu that fosters immunosuppressive cell infiltration (e.g. myeloid-derived suppressor cells, regulatory T cells), dampening cytotoxic T-lymphocyte activity and natural killer (NK) cell-mediated tumour clearance.^81^^,^^82^

Previous studies showed that the use of β-blockers can prolong progression-free survival in patients with different types of cancer,^83^ which indicates that β-adrenergic signalling in the SNS is a crucial regulator of cancer progression and metastasis, potentially becoming a target for future cancer therapy. In contrast, activation of PSNS delays tumour progression by inhibiting tumour stem cell activity. Experimental interventions such as VNS or AChE inhibitors restore parasympathetic tone, attenuate inflammation, and reduce tumour burden in animal models.^84^ However, the PSNS is abnormally innervated in gastrointestinal cancers. Cholinergic stimulation of the gastric epithelium induced nerve growth factor (NGF) expression, and, in turn, NGF overexpression within gastric epithelium expanded enteric nerves and promoted carcinogenesis.^85^ All these studies further indicate that perioperative ANS destruction, whether SNS or PSNS, may increase cancer recurrence.

### Perioperative organ dysfunction

Perioperative imbalance of ANS activity may be one of the objective causes of perioperative multiple organ failure, which may have adverse effects on the function of essential organs such as the heart, lung, immune system, and digestive system and eventually lead to the occurrence of MODS.

#### Cardiovascular dysfunction

Surgery and anaesthesia can trigger a sympathetic surge, leading to increased catecholamine release, which may result in hypertension, tachycardia, and increased myocardial oxygen demand causing myocardial ischaemia, particularly in patients with pre-existing coronary artery disease, leading to perioperative myocardial infarction.^86^ In contrast, perioperative autonomic imbalance, characterised by a relative reduction in parasympathetic tone, has been implicated in the development of arrhythmias, such as atrial fibrillation, which are common after major surgeries such as cardiac or thoracic procedures.^87^

#### Respiratory dysfunction

A reduction in parasympathetic tone, often seen with the use of anaesthetics such as propofol and neuromuscular blockers, can lead to bronchoconstriction and impaired gas exchange, further increasing the risk of postoperative pulmonary complications.^88^ In addition, perioperative hyperactivity of the SNS can increase pulmonary vascular resistance, leading to pulmonary hypertension and right ventricular dysfunction. This condition is particularly problematic in patients with pre-existing chronic obstructive pulmonary disease or pulmonary fibrosis, where the increased workload on the right heart results in heart failure.^89^

#### Renal and liver dysfunction

Kidneys are highly sensitive to changes in blood flow and oxygenation, both of which are influenced by the ANS. Perioperative sympathetic overactivity can lead to renal vasoconstriction, reduced renal blood flow, and decreased glomerular filtration rate, all of which contribute to the development of acute kidney injury (AKI).^90^ Similarly, the hepatic blood flow is tightly regulated by the balance between SNS and PSNS activity. An increase in sympathetic tone can reduce hepatic perfusion, leading to ischaemia and subsequent hepatocellular injury.^91^

#### Gut injury

The gastrointestinal tract is highly sensitive to changes in autonomic tone, particularly sympathetic overactivity, which can lead to splanchnic vasoconstriction and reduced mesenteric blood flow. This can cause intestinal ischaemia and reperfusion injury, compromising the integrity of the gut barrier and increasing the risk of bacterial translocation and systemic inflammatory response syndrome.^92^ Postoperative ileus, a common complication after abdominal surgery, is characterised by a temporary cessation of bowel motility. The use of opioids for postoperative pain management further contributes to ileus by inhibiting gastrointestinal motility through both central and peripheral mechanisms.^93^

#### Multiple organ dysfunction syndrome

Uncontrolled sympathetic activity during the perioperative period may drive cytokine release through multiple mechanisms, leading to MODS.^94^ ANS, as a marker of endothelial dysfunction and immunosuppression and multiple organ dysfunction, is closely associated with prolonged ICU stays and mortality in patients.^95^

## Perioperative autonomic nervous system rebalance

**Fig 4:**
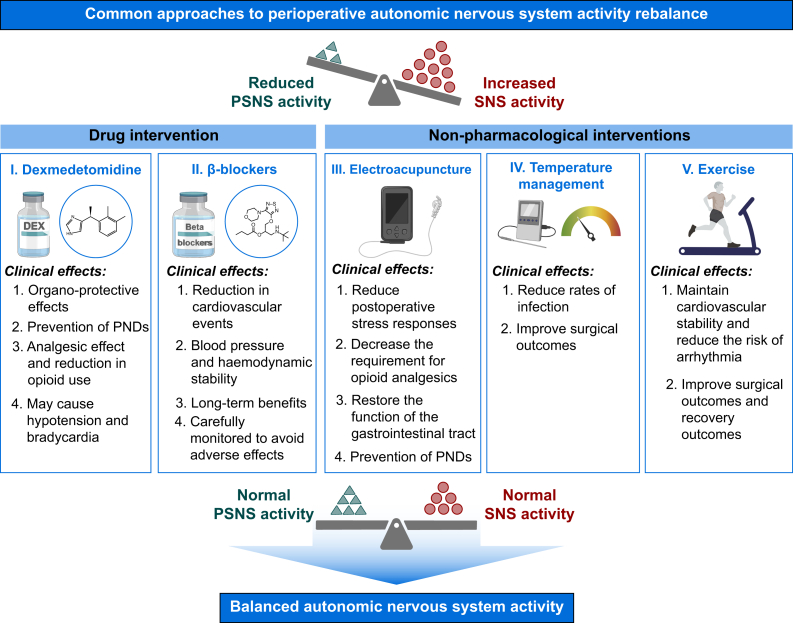
Treatment measures and related mechanisms of perioperative autonomic nervous system (ANS) tone rebalance. PND, postoperative neurocognitive disorders; PSNS, parasympathetic nervous system; SNS, sympathetic nervous system. Created with elements from https://BioRender.com.

Restoring balance of the ANS activity is imperative to enhance postoperative recovery and mitigate complications. Pharmacological interventions, including dexmedetomidine and β-blockers, alongside physical modalities such as electroacupuncture and temperature management, have been shown to effectively regulate the activity of both SNS and PSNS. These interventions attenuate the stress response, diminish the likelihood of cardiovascular complications, improve postoperative cognitive function, and enhance immune response (Fig. 4).

### Drug intervention

#### Dexmedetomidine

It is a selective α2-adrenergic receptor agonist with unique properties, providing sedation, analgesia, anxiolysis, anti-inflammatory effects, and cardiovascular stabilisation.^96^ It helps achieve ANS activity balance during perioperative period and reduces surgical stress responses while improving postoperative recovery.

Excessive sympathetic activity during surgery can lead to tachycardia, hypertension, and myocardial ischaemia, particularly in high-risk cardiovascular patients. In cardiac surgery, dexmedetomidine stabilises haemodynamic and lowers myocardial injury markers, improving cardiac function and reducing postoperative complications such as atrial fibrillation.^97^^,^^98^

Dexmedetomidine can reduce inflammatory response, reduce pulmonary microvascular hyperpermeability, improve pulmonary function, and prevent complications by activating α2-adrenergic receptors.^99^ Compared with propofol, dexmedetomidine improved recovery and prevented pulmonary complications.^100^ For patients with atelectasis and bronchial foreign body obstruction, dexmedetomidine combined with other drugs, such as nalbuphine, can enhance lung protection and promote patient recovery.^101^

Dexmedetomidine protects the liver by attenuating ischaemia–reperfusion injury during hepatectomy, reducing inflammatory cytokines and oxidative stress to preserve liver function and promoting better postoperative outcomes.^102^^,^^103^ In patients with cirrhosis, it preserves haemodynamic stability and mitigates stress responses while maintaining immune function, thereby facilitating recovery.^104^

In terms of renal protection, dexmedetomidine reduces postoperative AKI by activating α_2_-adrenergic receptors, enhancing the cell survival signal pAKT, suppressing HMGB1 release, and inhibiting TLR4 signalling.^105^ It also attenuates epithelial–mesenchymal transition and inhibits necroptosis via α_2_-adrenoreceptor activation in the renal tubular cells, preserving oxygenation and function.^106^ Additionally, dexmedetomidine can provide renal protection by activating the anti-inflammatory effect of PSNS.^107^

Dexmedetomidine significantly reduces postoperative delirium and neurocognitive dysfunction.^108^ In older noncardiac surgery patients, it lowers delirium risk,^109^ likely because of its sedative, anti-inflammatory, and cerebral blood flow-stabilising effects.^108^^,^^110^^,^^111^ Studies reported that its neuroprotection persists weeks post-surgery, mitigating long-term cognitive decline.^112^ Moreover, low-dose dexmedetomidine decreases cerebral hyperperfusion syndrome after carotid stenting by reducing middle cerebral artery flow,^113^ supporting its role in ANS regulation.

The clinical translation of the organ-protective effects of dexmedetomidine remains controversial. The DECADE trial, the largest RCT to date, paradoxically showed increased delirium risk.^114^ A Bayesian meta-analysis of 3539 cardiac surgery patients initially suggested reduced delirium incidence, but this benefit disappeared after adjusting for publication bias.^115^ Despite controversies, dexmedetomidine use demonstrated potential to modulate ANS tone and protected vital organs in high-risk states, highlighting its clinical significance when applied with individual patients.

#### β-blockers

β-blockers (β-adrenergic antagonists) are pivotal in perioperative cardiovascular management, reducing catecholamine-induced stress and stabilising haemodynamic, and improved surgical outcome, particularly in high-risk patients.^116^ Their ability to balance ANS activity and suppress excessive sympathetic activation also prevents long-term complications such as heart failure, recurrent ischaemia, and hypertensive crises.^117^

Perioperative β-blockers use lowered the incidence of myocardial infarction, particularly in high-risk patients undergoing noncardiac surgeries.^118^ This effect is mainly owing to their ability to reduce myocardial oxygen consumption by modulating the PSNS to reduce heart rate and contractility, and to improve coronary blood flow by reducing SNS activity and inhibiting adrenergic-mediated vasoconstriction.^119^ β-blockers help prevent perioperative arrhythmias by stabilising the myocardial electrical system, thus reducing the risk of life-threatening conditions such as atrial fibrillation and ventricular tachyarrhythmias, which often occur after surgical stress.^120^ Moreover, β-blockers play a crucial role in regulating perioperative blood pressure by mitigating the SNS excitation and catecholamine surge triggered by anaesthesia induction and surgical stimulation,^121^ contributing to controlled hypotension during surgery and averting extreme blood pressure drops that may lead to inadequate organ perfusion.^122^

Although β-blockers reduce catecholamine-induced stress and stabilise haemodynamic through ANS modulation, their net clinical benefits require careful risk stratification. Perioperative β-blockers use demonstrates a dual effect profile. The landmark is that patients undergoing noncardiac surgery (POISE) trial revealed a concerning trade-off: although myocardial infarction was reduced with β-blockers, both overall mortality and stroke risk were increased.^123^ This paradox may stem from their simultaneous modulation of both SNS and PSNS. During haemodynamic instability, excessive β-blockers can reduce myocardial oxygen demand by controlling heart rate but impair compensatory mechanisms. Contemporary evidence emphasises strict patient selection criteria. Current guidelines recommend continuation only in patients with pre-existing β-blocker indications, rather than *de novo* initiation before surgery. The cardiovascular protective effects against arrhythmias and hypertensive crises must be weighed against the POISE-identified risks.^124^

### Nonpharmacological interventions

#### Electroacupuncture

Perioperative electroacupuncture may rebalance ANS activity, improving patient outcomes by modulating pain perception through CNS and PSNS, promoting endogenous opioid and neuropeptide release.^125^ Studies indicate that electroacupuncture reduced postoperative nausea and vomiting and opioid use, particularly when applied to acupoints, likely owing to enhanced ANS balance.^126^ Additionally, electroacupuncture improves neurological and gastrointestinal function via the brain–gut axis, potentially preventing PND,^127^ while also reducing paralytic ileus incidence through increased parasympathetic tone and anti-inflammatory effects.^128^

#### Temperature management

Hypothermia, which is common during surgery, can severely impact the ANS by increasing sympathetic outflow and reducing parasympathetic activity, leading to increased blood loss, increased wound infection, and other poor prognosis.^129^ Use of active warming strategies, such as forced-air warming systems or heated i.v. fluids is recommended to preserve ANS activity balance and improve surgical outcomes.^130^

#### Exercise

Preoperative aerobic exercise enhances parasympathetic resilience, as measured by increased HRV and baroreflex sensitivity, thereby reducing postoperative atrial fibrillation and improving recovery outcomes. Previous studies demonstrated that tailored exercise programmes benefit patients with heart failure,^131^ while animal models reveal exercise-induced neuroplasticity in restoring vagal tone and baroreflex function in hypertensive rats.^132^ These findings suggest that incorporating aerobic exercise into preoperative care can be a valuable strategy for enhancing parasympathetic resilience and improving surgical outcomes.

## Conclusions

This review uniquely synthesises multidisciplinary evidence from anaesthesia, neuroscience, and immunology to mechanistically link surgical stressors and ANS-mediated pathophysiology. By integrating advanced assessment tools, we provide actionable insights for real-time monitoring of ANS dynamics, surpassing prior reviews focused on isolated biomarkers. Additionally, we dissect the effects of pharmacological interventions and nonpharmacological strategies, emphasising their potential to restore ANS equilibrium while addressing gaps in clinical translation. Therefore, targeted rebalancing of ANS tone is not merely a theoretical concept but a pragmatic pathway to preserve organ function and enhance patient recovery. Innovative monitoring and refined treatments to restore perioperative ANS balance hold promise for enhancing better surgical outcomes *per se*.

## Authors’ contributions

Conceptualisation: DM

Visualisation: WTP

Funding acquisition: JJY

Project administration: JJY, DM

Supervision: JJY, DM

Writing-original draft: WTP, MHJ

Writing-review and editing: JJY, DM

## Funding

National Natural Science Foundation of China (U23A20421 to JJY); British Journal of Anaesthesia; European Society of Anesthesiology and Intensive Care; and Ningbo Top Medial and Health Research Program (2024010317 to DM).

## Declaration of interest

The authors declare that they have no competing interests.
