# Supplementary material for: Effect of perioperative autonomic nervous system imbalance on surgical outcomes: a systematic review
Source: Br J Anaesth. 2025 Jul 4;135(3):608–22. doi: 10.1016/j.bja.2025.06.004 (PMC12489373; doi:10.1016/j.bja.2025.06.004)
Supplement: Multimedia component 1 [file mmc1.docx]

Online supplement for “Pan WT, et al., The effect of perioperative autonomic nervous system imbalance on surgical outcomes: A systematic literature review”

**Literature Search Strategy**

To ensure comprehensive identification of high-impact clinical trials and mitigate publication bias, a structured, multi-database search was conducted in accordance with PRISMA (Preferred Reporting Items for Systematic Reviews and Meta-Analyses) guidelines.

***Databases and Timeframe***

*Database****s*:** PubMed, Web of Science, EMBASE and Medline

*Timeframe:* A systematic search was conducted across PubMed, Embase, Web of Science, and Medline from January 2014 through April 2025. Key terms and concepts were identified through iterative scoping searches and consultation with subject matter experts. Boolean operators (AND/OR) and proximity searches were utilized to refine results.

***Timeframe selection reasons***

The rationale for choosing literature from 2014 to 2025 (although some key references were included beyond this period) is following:

First, key mechanistic studies on ANS-mediated pathophysiology, such as the cholinergic anti-inflammatory pathway, sympathetic-immune crosstalk, and neurochemical monitoring technologies emerged prominently after 2014.

Second, clinical trials and meta-analyses evaluating interventions like dexmedetomidine, β-blockers, and non-pharmacological strategies have been published within this period of time, reflecting evolving evidence for ANS rebalancing.

Third, recent innovations in real-time ANS assessment (e.g., acetycholine monitoring) and their clinical implications (e.g., postoperative neurocognitive disorders, cancer recurrence) necessitate focusing on contemporary studies to ensure relevance.

Finally, this time frame aligns with the authors’ aim to synthesize multidisciplinary insights (anaesthesia, neuroscience, immunology or other fields) that have matured significantly in the past decade, thereby strengthening our review to be clinical applicability and mechanistic depth. Studies that emerged during this period are not only limited to describing anatomical relationships and single functions, but also pay more attention to the connectivity and homeostasis maintenance between organs.

In addition, in the β-blockers section, the landmark trail (POISE trial, 2008) was retained despite out of the timeframe as it remains the cornerstone of evidence on perioperative β-blocker risks and benefits, with subsequent guidelines still referencing its findings.

***Search Terms (For example, PubMed)***

*Part 1 (ANS and homeostasis) and Part 2 (Perioperative pathophysiology and risk factors for ANS):*

("Autonomic Nervous System"[Mesh] OR "Parasympathetic Nervous System"[Mesh] OR "Sympathetic Nervous System"[Mesh]) AND ("Perioperative Period"[Mesh] OR "Postoperative Complications"[Mesh] OR low parasympathetic tone[Title/Abstract])

*Part 3 (Perioperative ANS function assessment):*

("Autonomic Nervous System"[Mesh]) AND ("Perioperative Period"[Mesh]) AND (pupillometry [Title/Abstract] OR heart rate variability [Title/Abstract] OR blood biomarkers [Title/Abstract] OR Electrodermal Activity[Title/Abstract])

*Part4 (Harmful postoperative ANS activity imbalance):*

("Autonomic Nervous System"[Mesh]) AND (neurocognitive disorders [Title/Abstract] OR immunosuppression [Title/Abstract] OR cancer recurrence [Title/Abstract] OR Cardiovascular dysfunction [Title/Abstract] OR Respiratory dysfunction [Title/Abstract] OR Gut dysfunction [Title/Abstract] OR Renal dysfunction [Title/Abstract] OR liver dysfunction [Title/Abstract])

*Part5 (Perioperative ANS rebalance):*

("Dexmedetomidine"[Mesh] OR "Adrenergic beta-Antagonists"[Mesh] OR "Electroacupuncture"[Mesh] OR "Vagus Nerve Stimulation"[Mesh] OR "Hypothermia, Induced"[Mesh]) AND ("Autonomic Nervous System"[Mesh])

***Inclusion and exclusion criteria***

Inclusion criteria prioritized original research articles, meta-analyses, and systematic reviews published in English that investigated ANS-mediated mechanisms or interventions in perioperative settings. Studies focusing solely on chronic autonomic disorders without perioperative relevance, case reports, and non-peer-reviewed publications were excluded.

***Extracted data***

Study design (RCT, cohort, animal model), sample size, surgical population (e.g., elderly, cardiac surgery), intervention details (e.g., Electroacupuncture acupoints and duration, Dose of dexmedetomine), ANS assessment methods (HRV indicators, blood biomarkers), and outcomes (e.g., complication rates, cytokine levels).

***Analysis***

The final synthesis integrated evidence from 132 studies (see below Supplemental Figure 1), encompassing molecular mechanisms, clinical observational data, and interventional trials. This multilayered approach ensured balanced representation of both narrative insights and systematic evidence, aligning with the review’s methodological framework.


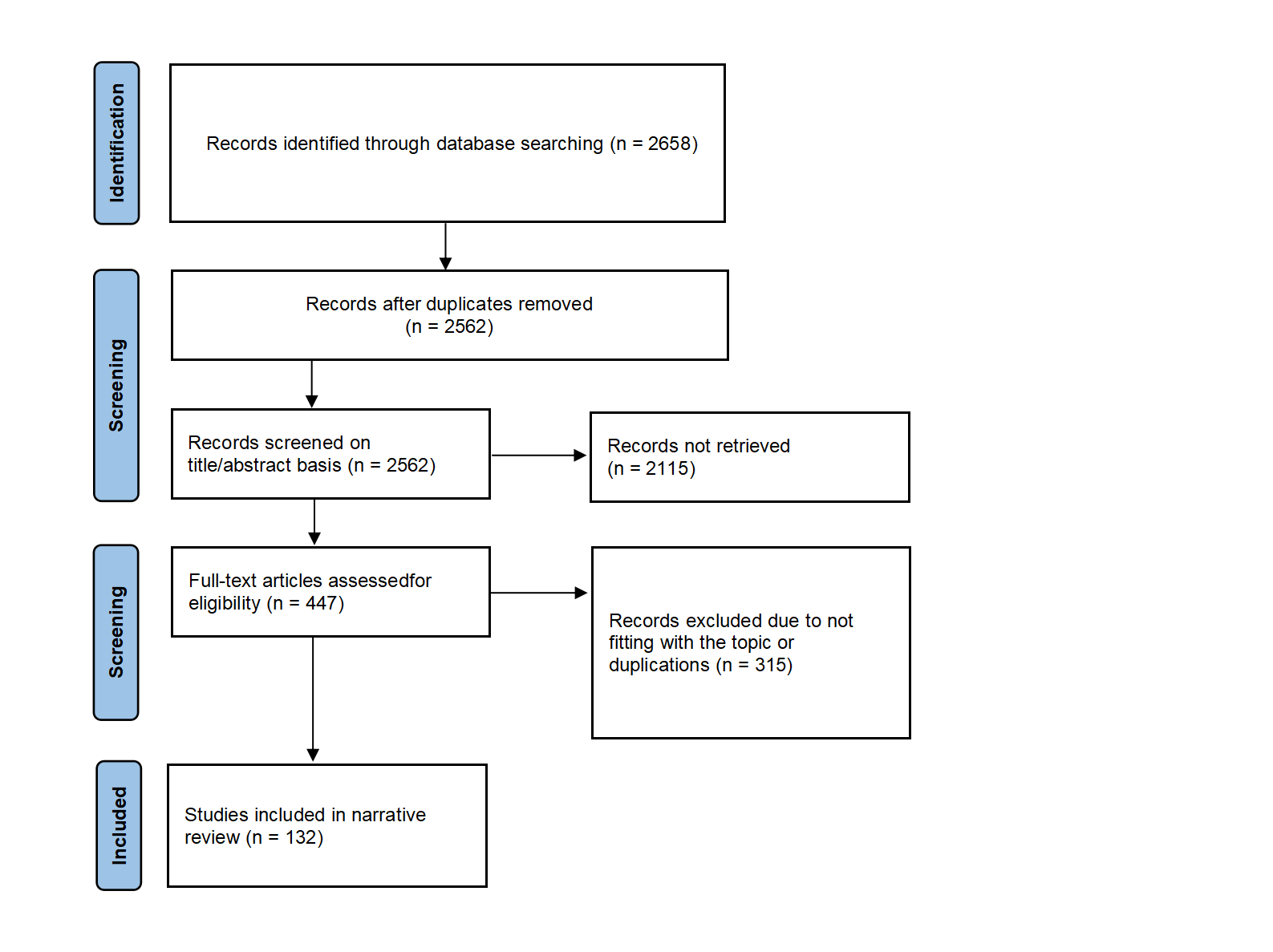


**Supplemental Figure 1.** Flow diagram of the literature screening process. Initially, 2,658 records were identified through database searching. After removing duplicates, 2,562 records remained. These were screened based on title and abstract, with 2,115 records not retrieved. A total of 447 full-text articles were then assessed for eligibility, of which 315 were excluded due to non-compliance with the topic or duplication. Finally, 132 studies were included in the narrative review.
